# Enhanced A_3 _adenosine receptor selectivity of multivalent nucleoside-dendrimer conjugates

**DOI:** 10.1186/1477-3155-6-12

**Published:** 2008-10-23

**Authors:** Athena M Klutz, Zhan-Guo Gao, John Lloyd, Asher Shainberg, Kenneth A Jacobson

**Affiliations:** 1 Molecular Recognition Section, Laboratory of Bioorganic Chemistry, National Institute of Diabetes and Digestive and Kidney Diseases, Bethesda, Maryland 20892, USA; 2 Mass Spectrometry Facility, Laboratory of Bioorganic Chemistry, National Institute of Diabetes and Digestive and Kidney Diseases, Bethesda, Maryland 20892, USA; 3 Faculty of Life Sciences, Bar-Ilan University, Ramat Gan, Israel

## Abstract

**Background:**

An approach to use multivalent dendrimer carriers for delivery of nucleoside signaling molecules to their cell surface G protein-coupled receptors (GPCRs) was recently introduced.

**Results:**

A known adenosine receptor (AR) agonist was conjugated to polyamidoamine (PAMAM) dendrimer carriers for delivery of the intact covalent conjugate to on the cell surface. Depending on the linking moiety, multivalent conjugates of the *N*^6^-chain elongated functionalized congener ADAC (*N*^6^-[4-[[[4-[[[(2-aminoethyl)amino]carbonyl]methyl]anilino]carbonyl]methyl]phenyl]-adenosine) achieved unanticipated high selectivity in binding to the cytoprotective human A_3 _AR, a class A GPCR. The key to this selectivity of > 100-fold in both radioreceptor binding (K_i app _= 2.4 nM) and functional assays (EC_50 _= 1.6 nM in inhibition of adenylate cyclase) was maintaining a free amino group (secondary) in an amide-linked chain. Attachment of neutral amide-linked chains or thiourea-containing chains preserved the moderate affinity and efficacy at the A_1 _AR subtype, but there was no selectivity for the A_3 _AR. Since residual amino groups on dendrimers are associated with cytotoxicity, the unreacted terminal positions of this A_3 _AR-selective G2.5 dendrimer were present as carboxylate groups, which had the further benefit of increasing water-solubility. The A_3 _AR selective G2.5 dendrimer was also visualized binding the membrane of cells expressing the A_3 _receptor but did not bind cells that did not express the receptor.

**Conclusion:**

This is the first example showing that it is feasible to modulate and even enhance the pharmacological profile of a ligand of a GPCR based on conjugation to a nanocarrier and the precise structure of the linking group, which was designed to interact with distal extracellular regions of the 7 transmembrane-spanning receptor. This ligand tool can now be used in pharmacological models of tissue rescue from ischemia and to probe the existence of A_3 _AR dimers.

## Background

Dendrimers bearing multiple ligands may have increased avidity to a receptor compared to the monovalent ligand, particularly if the ligand has a weak affinity for the receptor [1]. While this phenomenon has only been loosely demonstrated with PAMAM dendrimers, it is well established that multivalent oligo- and poly-saccharides, including PAMAM glycodendrimers, show some enhancement in binding compared to the monovalent saccharide, which is known as the cluster glycoside effect [2]. Dendrimer-ligand complexes have also been used as imaging agents [3] and for gene delivery [1]. Recently, we also attached CGS21680, an A_2A _adenosine receptor (AR) agonist, to G3 PAMAM dendrimers, providing the first example of a GPCR ligand to be conjugated covalently to a dendrimer while retaining its biological activity [4].

The ARs are GPCRs that have a generally cytoprotective role and their ligands are of increasing therapeutic interest. The A_1 _AR and A_3 _AR inhibit adenylyl cylase through the coupling of the G_i _protein and are also involved in activating phospholipase C and potassium channels [5]. The A_1 _AR is highly expressed in the brain, spinal cord, eye, and atria while intermediate expression is found in the liver, kidney, and adipose tissue [6]. The A_3 _AR is upregulated in peripheral blood mononuclear cells of patients with rheumatoid arthritis as well as in several breast, colon and pancreatic carcinoma tissues [7], but more studies are needed to learn about the expression of this protein in normal patients. Preconditioning of cardiomyocytes with either A_1 _or A_3 _AR agonists protects against myocardial ischemia. This cardioprotection occurs through extracellular signal-regulated kinase (ERK) signaling and activation of the mitochondrial K^+^-ATP channels [5]. A_1 _AR agonists also inhibit lipolysis [6] and may act as anti-epileptic agents [8], while A_3 _AR agonists may protect against lung injury and cancer [9,10].

**Figure 1:**
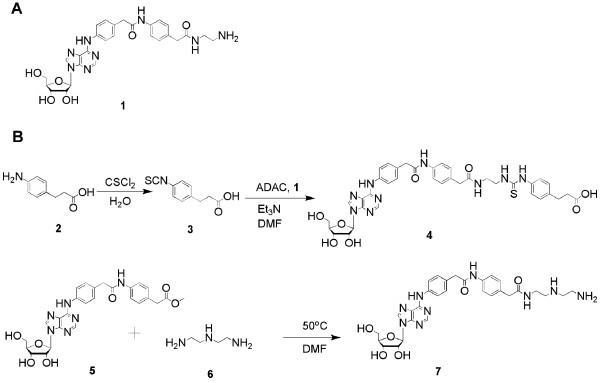
Synthesis of novel functionalized congener monomers related to ADAC.

The AR ligands chosen for conjugation to both G2.5 or G3 PAMAM dendrimers in the present study are the A_1 _AR agonist *N*^6^-[4-[[[4-[[[(2-aminoethyl)amino]carbonyl]methyl]anilino]-carbonyl]methyl]phenyl]adenosine (ADAC, **1**) and related functionalized congeners (Figure 1). Functionalized congeners are designed by adding a chain substituent to a pharmacophore in a strategic, permissive location so that conjugation to other large molecules is possible [11]. Ideally, the linker is modified to enhance the interaction of the pharmacophore with the receptor. This approach has been used to study A_1 _[12], A_2A _[13], and A_3 _ARs [11]. ADAC is a highly selective A_1 _AR agonist at the rat ARs and also displays some selectivity towards the human A_1 _AR and human A_3 _AR in comparison to the human A_2A _AR [5,14]. ADAC protects against neuronal damage and mortality after either acute or chronic administration prior to a ten-min bilateral cerebrovascular occlusion in gerbils. Significantly higher doses of other A_1 _AR agonists are needed to produce an equivalent effect [15]. ADAC also provides neuronal protection when given up to twelve hours post-ischemia [16]. Each of the dendrimer nucleoside conjugates also contained a fluorescent moiety for *in vitro *and *in vivo *localization.

## Results

This study was designed to probe the feasibility of modulating the potency and selectivity of nucleoside agonist ligands of ARs based on conjugation to a PAMAM nanocarrier.

### Synthesis of ADAC-Related Functionalized Congeners and Dendrimer Conjugates

ADAC, an amine-derivatized nucleoside that potently binds to and activates the A_1 _AR, was coupled covalently to the surface of polyamidoamine (PAMAM) dendrimers of generation 2.5 (G2.5). Two other linker moieties were applied for comparison: one containing a secondary amine and another containing an extended arylthiourea group, which was attached to a G3 PAMAM dendrimer as shown in Figure 1B. Two nucleoside intermediates related to ADAC, **4 **and **7**, which had chains that could be coupled to PAMAM dendrimers, were synthesized as shown in Figure 1. 3-(p-Aminophenyl)propanoic acid **2 **was converted to 3-(p-isothiocyanatophenyl)propanoic acid **3 **by addition of thiophosgene in aqueous medium. The isothiocyanate group of **3 **was then conjugated to the terminal amino group of ADAC to form a thiourea linkage in **4**, which had a terminal carboxyl group that could be coupled to the amino group of the G3 PAMAM dendrimer. To synthesize the diamino derivative **7**, diethylenetriamine **6 **was heated with methyl ester **5**, which was similar to a previous method [17]. This product has a terminal primary amine group that was coupled to the G2.5 PAMAM dendrimer, with preference for its acylation over the secondary amine.

**Figure 2:**
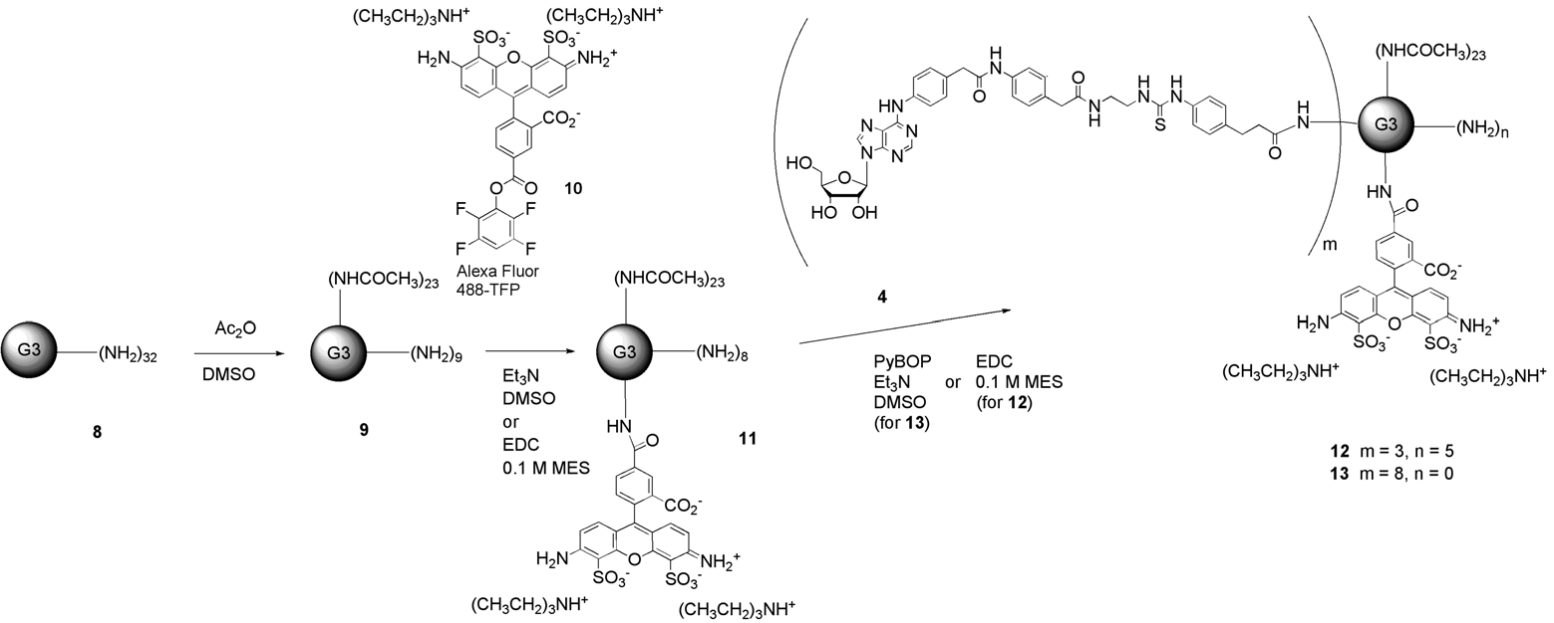
Synthesis of compounds 12 and 13 – derivatives of G3 PAMAM dendrimer.

Each of the G3 and G2.5 dendrimer conjugates also contained an AlexaFluor 488 (AF488) moiety [18] for fluorescent detection. G3-PAMAM-AF488-3 **4 **(**12**) and G3-PAMAM-AF488-8 **4 **(**13**) were synthesized as shown in Figure 2. First, the G3 dendrimer was partially acetylated with acetic anhydride to decrease toxicity. Next, the Alexa-Fluor 488 moiety was attached using either a PyBOP coupling in the presence of triethylamine as base [4] or an EDC coupling at pH 5 [19,20]. Finally, an amide bond was formed between the carboxyl group of **2 **and several terminal amines on the G3 dendrimer using a PyBOP coupling for **13 **[4] and an EDC coupling for **12 **[19,20].

Another goal was to compare 2.5 PAMAM – conjugates of A_1 _AR agonists with G3 PAMAM – conjugates of similar agonists. However, in order to attach AF488 to the carboxylic G2.5 dendrimer, it was necessary to synthesize a new AF488 derivative having a terminal primary amine. Initial attempts were made to couple ethylenediamine to **10 **using triethylamine in DMF or DMSO, but the AF488 did not appear stable under these conditions. However, a variation of the method using ethylenediamine in 0.1 M NaB_4_O_7_, pH 8.5, was successful. After HPLC purification and lyophilization, compound **14 **was isolated in 93% yield.

**Figure 3:**
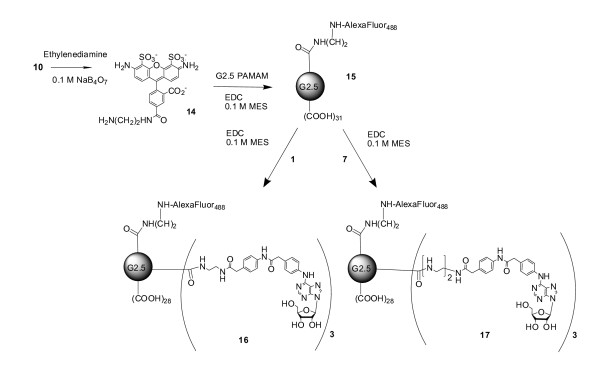
Synthesis of compounds 16 and 17 – derivatives of G2.5 PAMAM dendrimer.

G2.5-PAMAM-AF488-**1 **(**16**) and G2.5-PAMAM-AF488-**7 **(**17**) were synthesized as shown in Figure 3. First, a carbodiimide coupling was used to attach the AF488 derivative **14 **to the G2.5 dendrimer, using EDC in 0.1 M MES, pH 5 [19,20]. The unreacted EDC and urea byproduct were removed by dialysis. Next, the terminal amino groups of either **1 **or **7 **were amide conjugated to the G2.5 dendrimer also using a carbodiimide coupling.

The conjugates were purified using size exclusion chromatography and characterized using NMR and electrospray ionization (ESI) mass spectrometry (MS) (see Additional file 1). The parent G3 dendrimer matched its theoretical weight, but the parent G2.5 dendrimer appeared to be missing 2 propionate groups in the largest peak in the mass spectrum, as shown in Figure S1 (Additional file 1). Due to the excessive amount of sodium, the G2.5 spectrum was significantly more fragmented than the G3 spectrum. These spectra appear to be one of the first examples of using ESI MS rather than MALDI MS to obtain data on the PAMAM dendrimers.

After removal of the monomers by dialysis, NMR showed that approximately three and eight molecules of **4 **were attached per dendrimer, on average, in derivatives **12 **and **13**, respectively. Interestingly, while the mass spectrum of **13 **was very close to the theoretical mass, the mass spectrum of both compounds was very fragmented as shown in Figure S2 (Additional file 1). The largest peak of **12 **appeared to differ from the theoretical mass by approximately 1.8%, possibly due to the molecule breaking down in the mass spectrometer. The majority of the amino groups on the dendrimer appeared to be acylated, which has previously been shown to significantly decrease toxicity [21].

NMR indicated that on average there were approximately three nucleoside ligand moieties attached to each dendrimer in purified derivatives **16 **and **17**. The mass spectrum of **16 **was different from the theoretical mass by approximately 1 nucleoside moiety, possibly due to the compound decomposing in the mass spectrometer. The mass spectrum of **17 **was too fragmented to be useful as shown in Figure S3 (Additional file 1). The largest peak in the spectra was smaller than **15**, the dendrimer with only the AF488 moiety attached. However, smaller peaks in the spectrum were closer to the theoretical weight. Unlike the spectra for **13 **and **14**, there was significantly more sodium in these spectra, which may have caused the difficulties in obtaining these spectra. Also, the parent G2.5 dendrimer had more fragmentation than the parent G3 dendrimer. The difficulty in obtaining mass spectral data for dendrimers is a known phenomenon [21].

### Pharmacological Characterization of ADAC-Related Functionalized Congeners

The human AR binding affinity of these functionalized congeners was measured prior to attachment to the dendrimers (Table 1) [22]. Both **4 **and **7**, the new ADAC derivatives, had slightly lower affinity than ADAC itself at the A_1 _AR, with K_i app _values of 30 nM and 43 nM, respectively. While **4 **retained selectivity similar to ADAC towards the A_1 _AR in comparison to A_2A _AR, **7 **was slightly less selective. Interestingly, **7 **had a similar affinity for the A_3_AR as ADAC, while **4 **had significantly lower affinity at this receptor. [^35^S]GTPγS binding, a functional assay for G_i _protein activation [23], was completed in membranes expressing the A_1 _AR (Table 2). Compound **7**, with an EC_50 _value of 63 nM in activation of GTPγS binding via the A_1 _AR, was 20-fold more potent than **4 **and slightly more potent than ADAC. In an assay measuring the inhibition of the production of cAMP (Table 2), compound **7 **was also the most potent monomer at the A_1 _and A_3 _ARs. Compound **7 **was 3 – 7 fold more potent in adenylyl cyclase assays at these two ARs than either ADAC or **4**, which were nearly equipotent at both the A_1 _and A_3 _ARs. All compounds were shown to be full agonists at the A_1 _and A_3 _ARs in both assays.

**Table 1:** K_i _apparent values for binding of nucleoside monomers and dendrimer conjugates at A_1_, A_2A_, and A_3 _ARs.^a^

**Compound**	**A**_1 _K_i _(nM)	**A**_2A _K_i _(nM)	**A**_3 _K_i _(nM)
**Nucleoside Monomers**			
**1**	10.4 ± 3.8	370 ± 100	12.2 ± 4.1
**4**^**d**^	30 ± 9	800 ± 360	74 ± 20
**7**^**d**^	43 ± 5	300 ± 20	9.5 ± 2.0
**Dendrimer Derivatives**			
**11**	NB^b^	(20 ± 7%)^c^	(26 ± 3%)^c^
**12**	21 ± 5	250 ± 40	27 ± 2
**13**	55 ± 10	405 ± 170	42 ± 17
**15**	NB^b^	NB^b^	NB^b^
**16**	175 ± 60	610 ± 110	14.0 ± 2.1
**17**^d^	320 ± 20	470 ± 50	2.4 ± 0.4

a. All experiments were performed using adherent CHO cells stably expressing the A_1 _or A_3 _AR or HEK cells stably expressing A_2A _AR. Binding was carried out as described in methods using [^3^H]CCPA, [^3^H]CGS21680, or [^125^I]AB-MECA. Values are expressed as K_i _values (mean ± SEM, n = 3) or as displacement of the radioligand at 10 μM. b. NB, No binding. Inhibition of radioligand binding < 10% at 10 μM. c. Percent inhibition of radioligand binding at 10 μM. d. **4**, MRS5145; **7**, MRS5169; **17**, MRS5183.

**Table 2:** Functional EC_50 _values for nucleoside monomers and dendrimer conjugates to activate the A_1 _AR ([^35^S]GTPγS binding and cAMP inhibition) and A_3 _AR (cAMP inhibition).^a^

**Compound**	**A**_1 _ ([^35^S]GTPγS binding), EC_50 _(nM)	**A**_1_ (adenylyl cyclase), EC_50 _(nM)	**A**_3_ (adenylyl cyclase), EC_50 _(nM)
**Nucleoside Monomers**			
**1**	94 ± 26	400 ± 80	100 ± 50
**4**	1300 ± 400	350 ± 20	140 ± 70
**7**	63 ± 14	89 ± 17	36 ± 13
**Dendrimer Derivatives**			
**11**	50%^b^	inactive^c^	inactive^c^
**12**	190 ± 70	23 ± 10	25 ± 10
**13**	940 ± 70	54 ± 20	17 ± 2
**15**	< 10%^b^	inactive^c^	inactive^c^
**16**	2400 ± 1300	120 ± 1	14 ± 5
**17**	370 ± 190	260 ± 90	1.6 ± 0.4

a. All experiments were performed using adherent CHO cells stably expressing the A_1 _or A_3 _AR. Binding of [^35^S]GTPγS and assays using a cAMP kit were carried out as described in methods. Values are expressed as EC_50 _values (mean ± SEM, n = 3) or as displacement of the radioligand at 10 μM. b. Percent of [^35^S]GTPγS binding at 10 μM compared to full agonist. c. Compound produced less than 20% of total inhibition at 10 μM as seen by ADAC.

### Pharmacological Characterization of Nuceloside-Dendrimer (G3) Conjugates

In the radioligand binding studies, the G3 dendrimer-ligand conjugates **12 **and **13 **had a comparable affinity to the free monomer **4 **at the A_1 _AR, but maintained a lower degree of A_1 _selectivity compared to the A_2A _AR. However, both conjugates had a higher affinity at the A_3 _AR than the free monomer. The control dendrimer, **11**, which contained AF488 and multiple acetamide moieties but not the nucleoside ligand, showed no binding at the A_1 _AR. At the A_2A _and A_3 _ARs, weak binding inhibition was evident at 10 μM, which might be a result of association of the radioligand with the dendrimer conjugate at high concentrations. This phenomenon was seen in the A_2A _AR agonist-dendrimer conjugates as well [24]. The control dendrimer **11 **also showed slight activity at 10 μM in the stimulation of [^35^S]GTPγS binding. However, at 10 μM, **11 **was unable to significantly inhibit cAMP production at the A_1 _AR or the A_3 _AR. In an assay measuring [^35^S]GTYγS binding at the A_1 _AR, the G3 dendrimer ligand conjugates **12 **and **13 **had EC_50 _values that were at least 4 fold lower than the free monomer. Both of the dendrimer ligand conjugates **12 **and **13 **were almost 5 – 10 fold more potent at the A_1 _AR than the free monomer in an assay measuring inhibition of cAMP production. Therefore, conjugating the nucleoside **4 **to the dendrimer improved the potency in activation of the A_1 _AR even though the affinity was similar in the radioligand binding.

### Pharmacological Characterization of Nuceloside-Dendrimer (G2.5) Conjugates

**Figure 4:**
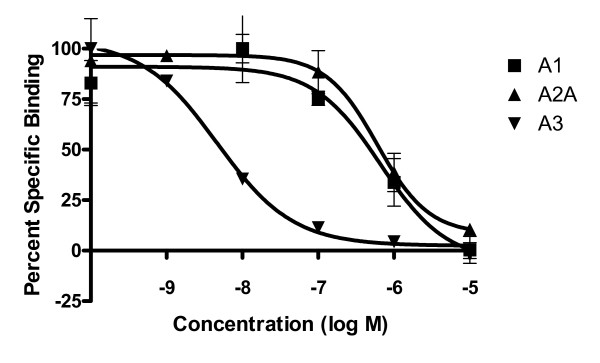
**Radioligand binding curves for 17.** Increasing concentrations of **17 **were incubated with the appropriate radioligand (A_1_: [^3^H]CCPA, A_2A_: [^3^H]CGS21680, A_3_: [^125^I]I-AB-MECA) and a suspension of CHO cell membranes (A_1 _or A_3_) or HEK cells (A_2A_) expressing the appropriate receptor. For a summary of K_i _values obtained, see Table 1.

**Figure 5:**
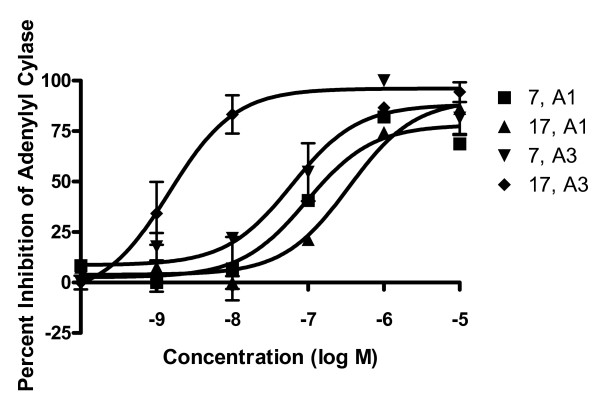
**cAMP inhibition curves for 7 and 17.** After 30 min incubation with increasing concentrations of **7 **or **17**, forskolin was added to CHO cells expressing A_1 _or A_3 _ARs to increase adenylyl cylase. The inhibition of adenylyl cylase was measured using the Direct cAMP Enzyme Immunoassay. For a summary of EC_50 _values obtained, see Table 2. The results shown are means ± S.E.M. of three independent experiments.

**Figure 6:**
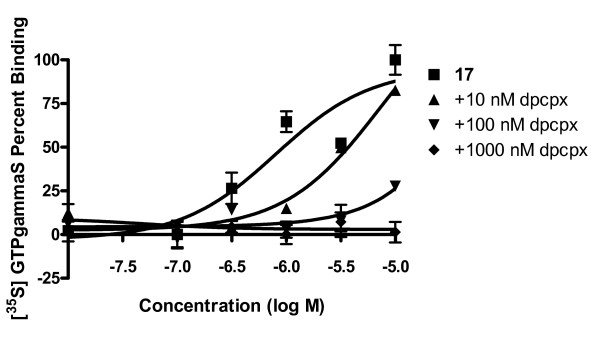
**Antagonism by an A_1_AR antagonist of [^35^S]GTPγS binding induced by compound **17**.** Compound **17 **was incubated with increasing concentrations of A_1 _antagonist DPCPX, [^35^S]GTPγS, and a CHO A_1 _membrane suspension. The amount of [^35^S]GTPγS bound was measured, and the results were interpreted with Prism software. The results shown are means ± S.E.M. of three independent experiments.

Radioligand binding was completed for each of the G2.5 dendrimer conjugates. Compound **17 **showed a 2.4 nM affinity for the A_3 _AR while compound **16 **had a 14 nM affinity for this receptor. Interestingly, **16 **displayed at least a 10-fold selectivity, and compound **17 **displayed over a 100 fold selectivity for the A_3 _AR in comparison to the A_1 _and A_2A _ARs (Figure 4). Compound **17 **was also 100 fold selective for the A_3 _AR in comparison to A_1 _AR in assays of adenylate cylase inhibition with an EC_50 _value of 1.6 nM at the A_3 _AR (Figure 5). However, in this assay, **16 **was only 8 fold more potent at the A_3 _AR than at the A_1 _AR. In GTPγS studies, **16 **was 15 fold less potent at the A_1 _AR than in an assay measuring the suppression of cAMP production; however, **17 **had similar potency at both A_1 _AR functional assays, and both compounds were full agonists in both assays. In the GTPγS study, DPCPX, an A_1 _antagonist, was able to fully inhibit the binding of [^35^S]GTPγS when incubated with **17 **(Figure 6), showing that the binding is due to the specific interaction of **17 **with the A_1 _receptor. The control dendrimer **15 **showed no binding or activity in either cAMP or GTPγS assays of A_1 _AR activation. The stably transfected CHO A_1 _and A_3 _cells had B_max _values of 530 ± 210 fmol/mg protein and 253 ± 19 fmol/mg protein, respectively, showing that there is similar receptor expression in both cell lines.

### Fluorescent Detection of Dendrimer (G2.5) Conjugates Bound to A_3 _AR Expressed in CHO Cells

**Figure 7:**
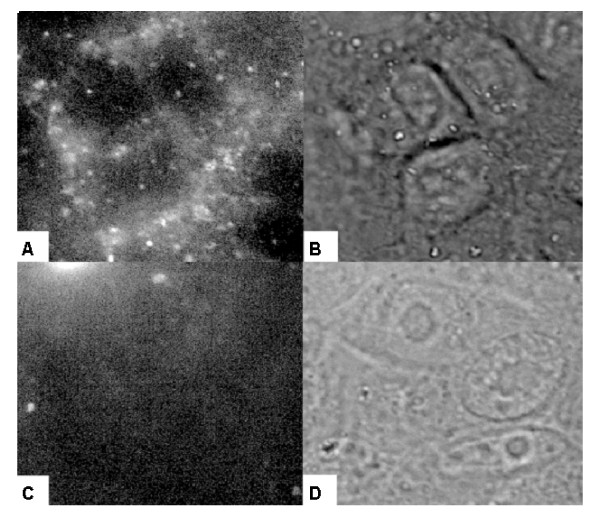
**Light and fluorescent microscopy of CHO or CHO A_3 _cells with compound **17**.** The cells were plated 24 h prior to the experiment. The cells were incubated for 1 h with the 10 μM of the appropriate compound and imaged with light and fluorescent microscopy. A. CHO A_3 _cells, fluorescent image; B. CHO A_3 _cells, light image; C. CHO cells, fluorescent image; D. CHO cells, light image.

10 μM of compounds **15 **or **17 **were incubated for 1 h with CHO cells that did or did not stably express the A_3 _AR. After one wash with PBS, the cells were imaged at 100× magnification on a Zeiss AxioVision D1 Imager, and both light and fluorescent pictures were obtained. As shown in Figure 7, only the cells expressing the A_3 _AR were bound by **17**. Neither type of CHO cells were bound by **15**, the control dendrimer with no ligand attached. **17 **was unable to bind CHO cells that did not express the A_3 _AR. While background fluorescence was seen for both compounds **15 **and **17 **when incubated with the CHO cells, this fluorescence did not correspond to the location of the cells. The fluorescence bound to the surface of the A_3_AR-expressing cells was not evenly distributed, but rather showed a punctuated distribution, possibly due to receptor aggregation.

## Discussion

Many drugs have already been delivered using dendrimers by bioreversible covalent conjugation, including methotrexate [25] and penicillin V [26]. Methotrexate, which was covalently attached via a hydrolyzable ester bond to a generation 5 (G5) PAMAM dendrimer that also contained folic acid, was significantly more toxic to folic acid receptor-expressing cancer cells than was the free ligand. The dendrimer with the ligand and folic acid attached and monomeric folic acid appeared to have a similar affinity for the folic acid receptor [25]. Our study differs from the bioreversible approach in that it describes covalent nucleoside-dendrimer conjugates that do not require cleavage in order to achieve a biological effect. It extends previous studies in which another receptor subtype, the A_2A _AR, was targeted [4,24].

The previous studies of A_2A _AR-directed dendrimers utilized exclusively amino-terminal dendrimers, such as G3 PAMAM dendrimers. Dendrimers with free amino groups at the periphery typically display a toxicity that is dependent on both size (i.e. generation) and concentration. This toxicity can be ameliorated by using neutral or anionic dendrimers, such as half-generation PAMAM dendrimers that have terminal carboxyl groups [21]. For instance, G3 PAMAM dendrimers caused significant haemolysis at 4 mg/ml in red blood cells, whereas G2.5 PAMAM did not cause haemolysis at 10 mg/ml [27]. In fact, only G7.5 PAMAM and higher generations caused significant haemolysis at 4 mg/ml. Therefore, we have included in this study dendrimers of half-generation, e.g. 2.5, which have terminal carboxylate groups and are therefore likely to be less toxic. When the G3 dendrimer was used in the present study, most of the amino groups were blocked to avoid cytotoxicity.

The differences between half-generation compared to integral generation PAMAM dendrimers in drug delivery have not been adequately studied. One study did attach methotrexate to G2.5 and G3 dendrimers using the terminal amine and carboxyl groups of the ligand, respectively, and the G2.5 conjugate had increased drug activity compared to either free methotrexate or the G3 conjugate [28]. However, the major reason given for the increased activity with the G2.5 derivative was that the methotrexate was released from the dendrimer due to prolonged interactions with proteases in the lysosome since the G2.5 derivative has an anionic charge. The paper concluded that it was probably necessary for the drug to be released from the dendrimer in order to retain its cytotoxic activity. However, GPCR ligands activate their receptors from outside of the cell, and it is unlikely that the ligand will need to be released from the dendrimer to retain activity. Therefore, there must be a different explanation for the improvement of selectivity and affinity of the G2.5 dendrimer-ligand conjugates.

The two functionalized congeners related to ADAC as well as ADAC itself were attached to the dendrimer through a terminal side chain attached to the same position on the nucleoside and which should not interfere with the binding of the adenosine moiety to the receptor. Each of the nucleoside monomers (compounds **1**, **4**, and **7**) showed less than a 5 fold difference in affinity between the human A_1 _and A_3 _ARs, but **17 **had an enhanced affinity and selectivity at A_3 _AR in both radioligand binding and cAMP assays. This enhancement can be explained by the difference in the linking moieties, such that the G2.5 dendrimer-ligand conjugates **16 **and **17 **allow a significant increase in selectivity towards the A_3 _AR compared to the A_1 _AR. This selectivity was not seen with the G3 dendrimer-ligand conjugates, which were approximately equipotent at the A_1 _and A_3 _ARs in both radioligand binding and cAMP assays. However, the decrease in affinity at the A_1 _AR and increase in affinity at A_3 _AR was seen in our previous work using A_2A _AR-directed G3 dendrimer-ligand conjugates. This work showed that dendrimer-nucleoside conjugates increased the selectivity at the A_2A _AR compared to the A_1 _AR by decreasing the binding affinity at the A_1 _receptor, and that all of the dendrimer ligand-conjugates were most potent at the A_3 _AR [24]. Dendrimer-conjugates **12 **and **13**, which are approximately equipotent at A_1 _and A_3 _ARs could be useful for cardioprotection [29], while A_3 _AR selective conjugates **16 **and **17 **could be useful in the treatment of rheumatoid arthritis [30].

Since residual amino groups on dendrimers are associated with cytotoxicity, the unreacted terminal positions of the A_3 _AR-selective G2.5 dendrimer **17 **were present as carboxylate groups, which had the further benefit of increasing water-solubility. Interestingly, not only does the A_3 _AR selectivity of **17 **improve upon conjugation to the dendrimer, but the affinity also slightly improves compared to the parent nucleoside, **7**. While this could be due to the fact that the nucleoside concentration is higher since there are multiple ligands attached per dendrimer, it is unlikely that this is the sole cause of the phenomenon. There are only on average three ligands attached per dendrimer, so it is unlikely that all of them are in the correct geometry to bind multiple receptor proteins in the membrane simultaneously. It is possible that the dendrimer-ligand conjugate would be blocking other receptor binding sites for the radioligand since it is much larger than the monomer. However, if this was the case, it would be expected that each of the dendrimer-ligand conjugates is more potent than the free nucleosides; instead, the conjugate **16 **is approximately equipotent to **1**. It could also be possible that due to the overexpression of the A_3 _AR on the CHO cells, A_3 _AR dimers are forming and the dendrimer conjugate is able to bridge the binding sites of both receptors. A_3 _ARs are known to accumulate in membrane microdomains and may form A_3 _AR homodimers [31]. If there is only one G protein associated with the GPCR dimer, the receptor that is not attached to the G protein could act as an anchor for the dendrimer ligand complex, allowing for a lowering of the EC_50 _and K_i app _values. Previously molecular modeling work at the A_2A _AR has shown that one dendrimer with multiple ligands could bridge an AR dimer [32]. However, further studies are necessary to elucidate the mechanism for the improvement of selectivity and potency of **17 **in comparison to **7**.

Compound **17 **was also studied using fluorescent microscopy. Interestingly, **17**, but not **15**, the control dendrimer with no ligand attached, was able to bind CHO cells expressing the A_3 _AR. Neither compound significantly bound to cells that were not expressing the A_3 _AR. The fluorescence remained associated with the cells expressing the A_3 _AR after washing. Compound **17 **appeared to bind some areas of the membrane more strongly than other areas as shown by increase in fluorescent signal. This finding could provide evidence that the A_3 _AR is condensed into patches on the cell membrane, possibly as dimers or oligomers. The uneven distribution of the fluorescent signal might indicate the existence of higher-order receptor oligomers, as it been recently demonstrated for the A_2A _AR [33]. We did not use transmission electron microscopy to determine if the fluorescent dendrimer conjugate was internalized by the cells. Internalization of other GPCRs under similar conditions, i.e., incubation with agonist for 1 hr at 37°C, is established. These two issues might be responsible of the punctuate distribution of A_3 _AR-dependent binding of **17**.

Compounds **12 **and **13 **are identical, except that **13 **contains an additional five ADAC moieties per dendrimer. Interestingly, attaching additional ADAC moieties to G3 PAMAM appeared to cause a slight decrease in affinity at all three ARs, although the selectivity remained similar. Our previous results comparing increasing numbers of A_2A _AR ligand attachments to dendrimers also failed to show a significant improvement in affinity by adding multiple ligands to the dendrimer [24]. However, in both of these studies, attaching the monomer to the dendrimer did not create a significant enhancement in affinity, unlike in our new G2.5 conjugates. Therefore, it will be interesting to determine if there is an enhancement in affinity when increasing numbers of ADAC moieties are attached to the G2.5 dendrimers.

## Conclusion

In conclusion, it is feasible to modulate and even enhance the pharmacological profile of a ligand of a GPCR based on conjugation to a nanocarrier and the precise structure of the linking group, which was designed to interact with distal extracellular regions of a 7 transmembrane-spanning receptor. We have demonstrated the feasibility of potent and selective activation of specific subtypes of ARs using multivalent conjugates and the ability to modulate the selectivity based on the linkage between the pharmacophore and the polymeric carrier. Both G2.5 and G3 PAMAM dendrimers can be successfully used in covalent dendrimer-ligand conjugates directed to GPCRs. High selectivity in binding at the A_3 _AR in comparison to the monomeric nucleosides could be achieved, depending on the nature of the linker moiety, i.e., a secondary amine linkage resulted in greater than 100-fold A_3 _AR selectivity. The selective macromolecular agonist **17 **can now be used in pharmacological models of tissue rescue from ischemia and as a fluorescent ligand tool to characterize the A_3 _AR *in situ *and to probe the existence of A_3 _AR dimers. Further studies will be completed using higher generation dendrimers and with new covalently-bound AR ligands. Other GPCRs may also be amenable to this approach to the design of multivalent ligands.

## Methods

### Materials

ADAC, PAMAM dendrimers (ethylenediamine core, generations 2.5 as 10 wt. % solution in methanol and generations 3 as 20 wt. % solution in methanol solution), 3-(4-aminophenyl)propionic acid, (benzotriazol-1-yloxy)tripyrrolidinophosphonium hexafluorophos-phate (PyBOP), 3-[(3-cholamidopropyl)dimethyl-ammonio]-1-propanesulfonate hydrate (CHAPS), adenosine deaminase, bovine serum albumin, sodium borate, guanosine 5'-diphosphate sodium salt (GDP), *N*-(3-dimethylaminopropyl)-*N*'-ethylcarbodiimide (EDC), dithiothreitol, ethylene-diaminetetraacetic acid acetic anhydride (EDTA), 2-(*N*-morpholino)ethanesulfonic acid (MES), magnesium chloride, sodium chloride, methanol, thiophosgene, triethylamine, diethyl ether, methyl sulfoxide-d_6 _(DMSO-d_6_), and *N*, *N*-dimethylformamide (DMF) were purchased from Sigma (St. Louis, MO). Bio-Beads^® ^SX-1 beads were purchased from Bio-Rad (Hercules, CA). Alexa-Fluor^® ^488 carboxylic acid, 2,3,5,6-tetrafluorophenyl ester, 5-isomer (AF488-TFP) was purchased from Invitrogen (Carlsbad, CA). [^125^I]-4-Amino-3-iodobenzyl-5'-*N*-methylcarboxamidoadenosine ([^125^I]AB-MECA, 2200 Ci/mmol), [^3^H]-2-chloro-N^6^-cyclopentyladenosine ([^3^H]CCPA, 42.6 Ci/mmol), and [^3^H]-2-[p-(2-carboxyethyl)phenylethylamino]-5'-N-ethylcarboxamidoadenosine ([^3^H]CGS21680, 40.5 Ci/mmol) were purchased from Perkin Elmer (Waltham, MA). [^35^S]GTPγS (1133 Ci/mmol) was purchased from GE Healthcare (Buckinghamshire, England). DMEM/F12 medium and 1 M Tris-HCl (pH 7.5) were purchased from Mediatech, Inc. (Herndon, VA).

### Chromatography and spectroscopy

To prepare a column for size exclusion chromatography (SEC), 100 g of Bio-Beads^® ^SX-1 beads were suspended in 1 L of DMF. After 24 h to allow for equilibration and expansion, the beads were added to the column as described previously [21]. High Performance Liquid Chromatography (HPLC) purification was performed using an Agilent 1100 Series HPLC (Santa Clara, CA) equipped with a Phenomenex Luna 5μ C18(2) 100A analytical column (250 × 10 mm; Torrance, CA). Peaks were detected by UV absorption using a diode array detector. Proton nuclear magnetic resonance spectra (NMR) were recorded on a Bruker DRX-600 spectrometer after being optimized for each sample using DMSO-d_6 _as a solvent unless otherwise noted. To determine the number of ligands attached to each dendrimer, the integration of NMR resonances of the ligand was compared to the integration of signal from one of the sets of carbon-protons on the interior of the dendrimer as described previously [4]. Electrospray ionization mass spectra (ESI MS) were taken using a Waters LCT Premier mass spectrometer. Matrix Assisted Laser Desorption/Ionization Time-of-Flight (MALDI-TOF) spectra were obtained with a Waters Micro mass spectrometer using Waters MassPREP Direct Ionization on Silica Desorption/ionization (DIOS) target plates.

The ESI MS data for the dendrimer complexes was obtained using a Waters LCT Premier TOF mass spectrometer. The mass spectrometer was operated in positive ion W mode with a resolution of 10000 measured at half peak height. The capillary voltage was 2500 volts, the cone voltage was 40 volts, and the desolvation gas was dried nitrogen at 250°C and a flow of 300 L/h. The sample was dissolved in a 1:1 solution of water:acetonitrile containing 0.2% formic acid and injected directly into the eluting stream flowing at 200 μL/min and consisting of 20:80 water:acetonitrile and 0.2% formic acid. The relevant spectra were summed using the MassLynx software, and the summed spectrum was deconvoluted with the MaxEntI program.

#### Chemical synthesis – 3-(4-Thiocarbamoylphenyl)propanoic acid (3)

3-(4-Aminophenyl)propanoic acid (**2**) (100 mg, 670 μmol) was dissolved in 0.7 mL of 0.8 M aqueous KOH. Thiophosgene (51.1 μL, 670 μmol) was diluted with 1.2 mL of water. The 3-(4-aminophenyl)propanoic acid solution was added dropwise to the freshly prepared thiophosgene solution. A solid immediately precipitated and redissolved upon the addition of 4.2 mL water. After 1 h, the solution was vacuum-filtered and vacuum-dried overnight to give 98.6 mg of 3-(4-thiocarbamoylphenyl)propanoic acid (475 μmol, 80% yield). ^1^H NMR (CDCl_3_) 7.27–7.30 (m, 2 H), 7.15–7.23 (m, 2 H), 2.93 (t, J = 7.9 Hz, 2 H), 2.68 (t, J = 7.4 Hz, 2H) *m/z *(M^+ ^ESI MS) calc: 208.0432 found: 208.0423.

#### 3-(4-(3-(2-(2-(4-(2-(4-(9-((2R,3R,4S,5R)-3,4-Dihydroxy-5-(hydroxymethyl)tetrahydrofuran-2-yl)-9H-purin-6-ylamino)phenyl)acetamido)-phenyl)acetamido)ethyl)thioureido)phenyl)pro-panoic acid (4)

3-(4-Thiocarbamoylphenyl)propanoic acid (**3**) (12 mg, 60.7 μmol) and ADAC (**1**) (35 mg, 60.7 μmol) were dissolved in 4 ml of DMF. Triethylamine (20 μL, 143 μmol) was added, and the reaction was stirred for 1 h. The DMF was removed under nitrogen and the resulting oil was dissolved in methanol. Ether was added to precipitate the product. After removal of the supernatant and drying, the resulting product (17.8 mg, 22.8 μmol, 37% yield) was judged homogenous by TLC. ^1^H NMR (DMSO-d_6_) 10.10 (s, 1H), 9.93 (s, 1H), 9.52 (br s, 1H), 8.53 (s, 1H), 8.37 (s, 1H), 8.10 (t, J = 6.1 Hz, 1H), 7.85 (d, J = 8.9 Hz, 2H), 7.66 (br s, 1H), 7.52 (d, J = 8.5, 2H), 7.21–7.33 (m, 4H), 7.13–7.20 (m, 4H), 5.95 (d, J = 6.0 Hz, 1H), 5.48 (d, J = 5.5, 1H), 5.29 (t, J = 5.6 Hz, 1H), 5.21 (d, J = 4.6 Hz, 1H), 4.63 (m, 1H), 4.17 (m, 1H), 3.98 (dd, J = 3.6, 4.0 Hz, 1H), 3.65–3.74 (m, 1H), 3.59 (s, 1H), 3.53 (m, 2H), 3.20–3.29 (m, 2H), 2.72–2.83 (m, 2H) *m/z *(M^+ ^ESI MS) calc: 784.2877 found: 784.2882.

#### N-(2-(2-Aminoethylamino)ethyl)-2-(4-(2-(4-(9-((2R,3R,4S,5R)-3,4-dihydroxy-5-(hydroxymethyl)-tetrahydrofuran-2-yl)-9H-purin-6-ylamino)-phenyl)acetamido)phenyl)acetamide (7)

This compound was synthesized according to a similar procedure to obtain ADAC (17).*N*^6^-[4-[[[4-((2-methoxy)-2-oxyethyl)anilino]carbonyl]-methyl]-phenyl]adenosine (**5**) (4.97 mg, 9.1 μmol) was dissolved in 1 mL of DMF, and diethylenetriamine (**6**) (150 μL, 1.37 mmol) was added to this solution. The reaction was stirred overnight under nitrogen, and the DMF was removed under a stream of dry nitrogen. The resulting oil was dissolved in methanol, and a solid was precipitated upon addition of ether. After removal of the supernatant, the remaining solid was dried overnight to give 3.75 mg of product (6.05 μmol, 66.5% yield). ^1^H NMR (DMSO-d_6_) 10.11 (s, 1H), 9.94 (br s, 1H) 8.53 (s, 1H), 8.38 (s, 1H), 7.90–8.04 (m, 2H), 7.84 (d, J = 9.0 Hz, 2H), 7.51 (d, J = 7.6 Hz, 2H), 7.29 (d, J = 8.4 Hz, 2H), 7.18 (d, J = 8.8 Hz, 2H), 5.95 (d, J = 6.9 Hz, 1H) 5.32 (m, 1H), 4.63 (t, J = 5.7 Hz, 1H), 4.17 (t, J = 4.8 Hz, 1H), 3.98 (dd, J = 3.4 Hz, 2.1Hz, 1H), 3.59 (m, 3H), 3.10–3.15 (m, 4H), 2.62 (m, 2H), 2.55 (s, 6H, 21, 22), 1.10 p (t, J = 6.8 Hz, 3H). *m/z *(M^+ ^ESI MS) calc: 620.2945 found: 620.2931.

#### 2-(6-Amino-3-iminio-4,5-disulfonato-3H-xanthen-9-yl)-5-(2-aminoethylcarbamoyl)benzoate (14)

5 mg of AF488-TFP (**10**) (5.65 μmol) was dissolved in 280 μL of DMF. In order to provide a basic environment, 1.50 mL of 0.1 M NaB_4_O_7_, pH 8.5 was added. 10 μL of ethylenediamine diluted with 210 μL of water was added, and the mixture was stirred overnight. The product was purified by HPLC using the following water/acetonitrile linear gradient: 0 min, 0% acetonitrile; 25 min, 100% acetonitrile. The product eluted at 10.8 min. After lyophilization, 3.02 mg of product (5.2 μmol) remained (93% yield). ^1^H NMR (D_2_O) 8.33 (d, J = 2.3 Hz, 1H), 8.17 (t, J = 1.9 Hz, 1H), 7.90 (m, 1H), 7.24 (t, J = 7.5, 1H), 7.16 (dd, J = 7.5, 1.9, 1H), 7.08 (d, J = 9.6, 2H), 6.83 (t, J = 8.8, 4H), 3.63 (m, 1H), 3.51 (t, J = 11.7, 2H), 3.40 (t, J = 5.0, 2H), 3.13–3.21 (m, 2H)*m/z *(M^- ^Na MALDI-TOF MS) calc: 597.0362 found: 597.0383.

#### G3 PAMAM – 23 Ac – AF488 (11) – Method 1

1 mL of G3 PAMAM methanol stock solution (18.8 mM, 18.8 μmol, Sigma) was added to a flask, and the methanol was evaporated. The remaining polymer was dissolved in 1 mL of DMSO-d_6_. Acetic anhydride (40.8 μL, 432 μmol, 23 eq) was diluted in 1 mL of DMSO-d_6_, and this solution was added dropwise to the solution of G3 PAMAM while stirring. After 18 h, an NMR spectrum showed approximately 23 acetamide groups per dendrimer, as expected, to give **9**. 460 μl of this solution (4.32 μmol, 9.4 mM) was removed and diluted to 1.46 mL with DMSO-d_6_. Triethylamine (10 μL, 72 μmol) was added under a nitrogen atmosphere. AF488-TFP (**10**) (4 mg, 4.52 μmol, 1.05 eq) was dissolved in 400 μL of DMSO-d_6 _and added to the mixture. After 48 h, the solution was vacuum filtered to remove a small amount orange precipitate that formed. The NMR spectrum was consistent with the assigned structure, but the signals resulting from AF488 could not be properly integrated due to the large G3 PAMAM peaks. The molecular weight of the compound was unable to be determined using either ESI or MALDI-TOF MS. Therefore, it was assumed that approximately one Alexa-Fluor 488 moiety was attached per G3 PAMAM, based on previous data [4].

#### G3 PAMAM – 23 Ac – AF488 (11) – Method 2

0.5 mL of G3 PAMAM methanol stock solution (18.8 mM, 9.4 μmol) was added to a flask, and the methanol was evaporated. The remaining polymer was dissolved in 0.5 mL of DMSO-d_6_. Acetic anhydride (20.4 μL, 216 μmol, 23 eq) was diluted in 0.5 mL of DMSO-d_6_, and this solution was added dropwise to the solution of G3 PAMAM with stirring. After 18 h, an NMR spectrum of the reaction mixture showed approximately 23 acetamide groups per dendrimer, as expected, to give **9**. 234 μL of this solution (2.2 μmol, 9.4 mM) was removed and diluted to 500 μL with DMSO-d_6_. AF488-TFP (**10**) (2 mg, 2.3 μmol, 1.05 eq) was dissolved in 300 μL of 0.1 M MES, pH 5 and added to the mixture under nitrogen atmosphere. EDC (42 mg, 220 μmol) was dissolved in 300 μL of 0.1 M MES, pH 5 and added to the reaction mixture. After 48 h, the solution was vacuum filtered to remove a small amount orange precipitate that formed. The NMR spectrum was consistent with the assigned structure, but the signals resulting from AF488 could not be properly integrated due to the large G3 PAMAM peaks. The molecular weight of the compound was unable to be determined using either ESI or MALDI-TOF MS. Therefore, it was assumed that approximately one Alexa-Fluor 488 moiety was attached per G3 PAMAM based on previous data [4].

#### G3 PAMAM – 23 Ac – AF488 (containing 3 moieties of 4) (12)

650 μL of **11 **prepared by method 2 (2.0 mM solution in DMSO-d_6_, 1.3 μmol) was removed and placed under nitrogen gas. Compound **4 **(5 mg, 6.4 μmol) was dissolved in 600 μL of DMSO and added to the mixture under a nitrogen atmosphere. EDC-HCl (25 mg, 130 μmol) was dissolved in 350 μL 0.1 M MES, pH 5 and added to the mixture. After 48 hr, the product was purified by extensive dialysis (Specta/Por Membrane, MWCO 3500, flat width 18 mm, Spectrum Laboratories, Inc., Rancho Dominguez, CA) in water. After lyophilization, 5.85 mg remained, which contained on average 3 moieties of **4 **per dendrimer (0.54 μmol, 41% yield based on μmol of dendrimer). 10.11 (s, 4H), 9.94 (s, 3H), 8.54 (s, 4H), 8.38 (s, 4H), 8.00 (s, 45H), 7.92 (s, 41H), 7.85 (s, 13H), 7.51 (br s, 6H), 7.29 (d, J = 7.5, 9H), 7.17 (m, 6H), 5.96 (d, J = 6.1, 2H), 5.49 (d, J = 4.2, 2H), 5.30 (t, J = 4.8, 2H), 5.22 (m, 3H), 4.63 (m, 3H), 4.18 (m, 3H), 3.98 (m, 3H), 3.33 (s, 226H), 3.08 (s, 203H), 2.70 (s, 124H), 2.23 (s, 120H), 1.80 (s, 69H). *m/z *(M^+ ^ESI MS) calc: 10865 found: 11068.

#### G3 PAMAM – Ac – AF488 AF488 (containing 8 moieties of 4) (13)

660 μL of **11 **prepared by method 1 (2.3 mM solution in DMSO-d_6_, 1.52 μmol) was removed and placed under nitrogen gas. Compound **4 **(11.8 mg, 15 μmol) was dissolved in 200 μl of DMSO and added to the mixture. Finally, a mixture of triethylamine (28 μL, 202 μmol) and PyBOP (26 mg, 50 μmol) dissolved in 1.5 mL of DMSO was added. After 48 hr, the product was purified by SEC using DMF as the eluent. The fractions containing product which had the Alexa-488 moiety were dried and dissolved in DMSO-d_6 _for NMR. The first and last fractions containing the product were excluded to provide a more homogenous sample. The remaining fractions were combined and dried to give 8.68 mg of product, which contained on average 8 moieties of **4 **per dendrimer (0.694 μmol, 46% yield based on μmol of dendrimer). ^1^H NMR (DMSO-d_6_) 10.11 (s, 8H), 9.94 (s, 8H), 9.53 (s, 5H), 8.54 (s, 8H), 8.38 (s, 8H), 8.10 (t, J = 6.0 Hz), 7.96 (s, 41H), 7.90 (s, 33H), 7.84 (m, 41H), 7.52 (d, J = 8.6 Hz, 16H), 7.24 (d, J = 8.3 Hz, 16H), 7.17 (m, 34H), 5.96 (d, J = 6.2, 7H), 5.50 (br s, 4H), 5.31 (br s, 7H), 5.22 (br s, 5H), 4.64 (t, J = 4.2 Hz, 8H), 4.18 (t, J = 3.4 Hz, 8H), 3.99 (dd, J = 2.7, 3.7, 8H), 3.70 (m, 11H), 3.59 (m, 44H), 3.08 (s, 176H), 2.90 (s, 8H), 2.74 (s, 9H), 2.65 (s, 120H), 2.43 (s, 77H), 2.19 (s, 120H), 1.80 (s, 69H). *m/z *(M^+ ^ESI MS) calc: 14241 found: 14226.

#### G2.5 PAMAM – AF488 (15)

This procedure was adapted from a carbodiimide coupling described previously [19,20]. 5 μmol of G2.5 PAMAM stock solution (14.3 mM in methanol, 31.3 mg) was added to a flask, and the methanol was evaporated. The remaining residue containing the polymer and compound **14 **(3.0 mg, 5.2 μmol) were dissolved in 1.7 mL of 0.1 M MES buffer, pH 5. EDC (40.4 g, 260 μmol) dissolved in 1 mL of 0.1 M MES buffer, pH 5, was added, and the reaction stirred for 60 h. After dialysis with water, the mixture was lyophilized to give 13.4 mg (1.97 μmol, 37% yield) and redissolved in D_2_O for NMR measurements and further biological assays. ^1^H NMR (D_2_O) 8.25 (s, 1H), 7.78–8.10 (m, 1H), 7.53 (s, 1H), 7.32 (s, 1H), 7.10 (s, 1H), 6.89 (m, 1H), 3.42 (s, 26H), 3.15 (m, 90H), 2.72 (s, 120H), 2.52 (s, 60H), 2.38 (m, 122H). The molecular weight was unable to be determined using ESI or MALDI-TOF MS due to stacking of the PAMAM dendrimer.

#### G2.5 PAMAM – AF488 (containing 3 moieties of 1) (16)

620 μL of a stock solution of **15 **in D_2_O was dried to give 9.32 mg (1.4 μmol), which was redissolved in 1 mL of 0.1 M MES, pH 5 and placed under a nitrogen atmosphere [20]. ADAC (8.92 mg, 14 μmol) was dissolved in 600 μL of DMSO and was added to the solution of **15**. Finally, 27 mg of EDC (141 μmol) was dissolved in 500 μL of 0.1 M MES, pH 5 and added to the mixture. After approximately 48 h, small molecule impurities were removed by extensive dialysis in water. After lyophilization, 8.06 mg (0.96 μmol, 68% yield) of product remained. The product was analyzed by NMR, which showed approximately 3 ADAC moieties attached per dendrimer. ^1^H NMR (DMSO-d_6_) 8.48 (s, 3H), 8.35 (s, 3H), 7.76 (s, 6H), 7.47 (m, 6H), 7.29 (m, 6H), 7.16 (m 6H), 5.92 (d, J = 5.5, 3H), 4.60 (m, 3H), 4.16 (m, 3H), 3.99 (m, 3H), 3.56 (s, 31H), 3.07 (s, 90H), 2.63 (s, 120H), 2.44 (s, 70 H), 2.20 (s, 120H). *m/z *(M^+ ^ESI MS) calc: 8405 found: 7811

#### G2.5 PAMAM – AF488 (containing 3 moieties of 7)(17)

640 μL of a stock solution of **15 **in D_2_O was dried to give 10.2 mg (1.5 μmol), which was redissolved in 600 μL of 0.1 M MES, pH 5 and placed under a nitrogen atmosphere. **7 **(9.2 mg, 15 μmol) was dissolved in 1 mL of DMSO and was added to **15**. Finally, 28 mg of EDC (146 μmol) was dissolved in 500 μL of 0.1 M MES, pH 5 and added to the mixture. After approximately 48 h, small molecule impurities were removed by extensive dialysis in water. After lyophilization, 9.74 mg (1.14 μmol, 76% yield) of product remained. The product was analyzed by NMR, which showed approximately 3 ADAC moieties attached per dendrimer. ^1^H NMR (DMSO-d_6_) 10.20 (s, 2H), 9.86 (s, 2H), 8.47 (s, 2H), 8.35 (s, 2H), 7.78 – 8.30 (m, 91H), 7.76 (br s, 7H), 7.47 (br s, 6H), 7.29 (br s, 5H), 7.16 (br s, 6H), 5.92 (t, J = 5.7, 2H), 5.30 (m, 6H), 4.60 (m, 5H), 3.54 (s, 46H), 3.10 (s, 104H), 2.98 (m, 28H), 2.63 (s, 160H), 2.42 (s, 78 H), 2.36 (s, 56H), 2.20 (s, 120H). The mass spectrum of this compound was too fragmented to determine a molecular weight.

### Cell Culture and Membrane Preparation

CHO (Chinese hamster ovary) cells stably expressing the recombinant human ARs were cultured in Dulbecco's modified Eagle medium (DMEM) and F12 (1:1) supplemented with 10% fetal bovine serum, 100 units/mL penicillin, 100 μg/mL streptomycin, and 2 μmol/mL glutamine. After harvesting, cells were homogenized and suspended. Cells were then centrifuged at 500 *g *for 10 min, and the pellet was resuspended in 50 mM Tris-HCl buffer (pH 7.5) containing 10 mM MgCl_2_. The suspension was homogenized and was then recentrifuged at 20 000 *g *for 20 min at 4°C. The resultant pellets were resuspended in Tris buffer, incubated with adenosine deaminase for 30 min at 37°C, and the suspension was stored at -80°C until the binding experiments. The protein concentration was measured using the BCA Protein Assay Kit from Pierce [20].

### Radioligand Membrane Binding Studies

Radioligand binding assays were performed for A_1 _and A_2A _ARs, following the procedure described previously [22]. Each tube in the binding assay contained 100 μL of membrane suspension (20 μg of protein), 50 μL of agonist radioligand, and 50 μL of increasing concentrations of the test ligands in Tris-HCl buffer (50 mM, pH 7.5) containing 10 mM MgCl_2_. The concentration of the dendrimer-ligand complexes are measured by the concentration of the dendrimer, not the ligand. Therefore, all K_i _values are measured as K_i app _values. Nonspecific binding was determined using a final concentration of 10 μM 5'-*N*-ethylcarboxamidoadenosine diluted with the buffer. The mixtures were incubated at 25°C for 60 min. Binding reactions were terminated by filtration through Whatman GF/B filters under a reduced pressure using a MT-24 cell harvester (Brandell, Gaithersburg, MD). Filters were washed three times with 5 mL of 50 mM ice-cold Tris-HCl buffer (pH 7.5). The radioactive agonists [^3^H]2-chloro-*N*^6^-cyclopentyladenosine and [^3^H]2-(4-(2-carboxyethyl)phenylethylamino)-5'-*N*-ethylcarboxamido-adenosine were used for the A_1 _and A_2A _assays, respectively. All of the filters were washed 3 times with Tris-HCl, pH 7.5. Filters for A_1 _and A_2A _AR binding were placed in scintillation vials containing 5 mL of Hydrofluor scintillation buffer and counted using a Perkin Elmer Liquid Scintillation Analyzer. Filters for A_3 _AR binding were counted using a Packard Cobra II γ-counter. The K_i _values were determined using GraphPad Prism for all assays.

### cAMP Assays

CHO cells expressing either the A_1 _or A_3 _AR were seeded in 24 well plates and incubated at 37°C overnight. The following day the medium was removed and replaced with DMEM containing 50 mM HEPES, 10 μM rolipram, 3 U/ml adenosine deaminase and increasing concentrations of the compounds. After a 30 min incubation at 37°C, 10 μM of forskolin was added to stimulate cAMP levels, and the cells were incubated at 37°C for an additional 15 min. The medium was removed, and the cells were lysed with 200 μl of 0.1 M HCl. 100 μl of the HCl solution was used in the Sigma Direct cAMP Enzyme Immunoassay following the instructions provided with the kit. The results were interpreted using a Bio-Tek Instruments ELx808 Ultra Microplate reader at 405 nm.

### [^35^S]GTPγS Binding Assay

[^35^S]GTPγS binding was measured in 200 μl of buffer containing 50 mM Tris-HCl (pH 7.4), 1 mM EDTA, 1 mM MgCl_2_, 10 μM GDP, 1 mM dithiothreitol, 100 mM NaCl, 3 units/ml adenosine deaminase, 0.2 nM [^35^S]GTPγS, 0.004% CHAPS, 0.5% bovine serum albumin and increasing concentrations of the ligands. Samples were started by addition of the membrane suspension (5–10 μg protein/tube) to the test tubes and incubated at 25°C for 30 min. The assay was terminated by rapid filtration through Whatman GF/B filters, pre-soaked in 50 mM Tris-HCl (pH 7.4) containing 5 mM MgCl_2 _and 0.02% CHAPS. Non-specific binding of [^35^S]GTPγS was measured in the presence of 10 μM unlabelled GTPγS. After the filters were washed, they were placed in scintillation vials containing 5 mL of Hydrofluor scintillation buffer and counted using a Perkin Elmer Liquid Scintillation Analyzer. The EC_50 _values were determined using GraphPad Prism for all assays [23].

### Light and Fluorescent Microscopy

CHO or CHO A_3 _cells were seeded on a cover disk in a 6 well dish (250,000 cells per well). After the cells were incubated for 24 hrs at 37°C, the medium was removed and replaced with DMEM containing 10 μM of the **15 **or **17**. The cells were incubated for 1 h at 37°C and washed one time with PBS. The images were taken with at 100× manification using a Zeiss AxioVision D1 Imager equipped with AxioVision 4.5 software.

## Abbreviations

ADAC: *N*^6^-[4-[[[4-[[[(2-aminoethyl)amino]carbonyl]methyl]-anilino]carbonyl]methyl]phenyl]adenosine; AF488-TFP: Alexa-Fluor^® ^488 carboxylic acid, 2,3,5,6-tetrafluorophenyl ester, 5-isomer; AR: adenosine receptor; CHAPS: 3-[(3-cholamidopropyl)dimethylammonio]-1-propanesulfonate hydrate; CHO: Chinese hamster ovary; DMEM: Dulbecco's Modified Eagle Media; DMF: *N, N*-dimethylformamide; DMSO: dimethyl sulfoxide; EDC: *N*-(3-dimethylaminopropyl)-*N*'-ethylcarbodiimide; EDTA: ethylenediaminetetraacetic acid; ERK: extracellular signal-regulated kinase; ESI: electrospray ionization; GDP: guanosine 5'-diphosphate; GPCR: G protein-coupled receptor; [^3^H]CCPA: 2-chloro-*N*^6^-cyclopentyladenosine; [^3^H]CGS21680: 2-[*p*-(2-carboxyethyl)phenylethylamino]-5'-*N*-ethylcarboxamido-adenosine; HEK: human embryonic kidney; HEPES: 4-(2-hydroxyethyl)-1-piperazineethanesulfonic acid; [^125^I]AB-MECA: [^125^I]-4-aminobenzyl-5'-*N*-methylcarboxamideoadenosine; MALDI-TOF: matrix assisted laser desorption/ionization time-of-flight; MES: 2-(*N*-morpholino)ethanesulfonic acid; MS: mass spectrometry; NMR: nuclear magnetic resonance; PAMAM: poly(amidoamine); PyBOP: benzotriazol-1-yl-oxytripyrrolidinophosphonium hexafluorophosphate.

## Competing interests

KAJ and AMK are listed as inventors on a related patent application assigned to the Department of Health and Human Services.

## Authors' contributions

AMK did the pharmacological assays, chemical synthesis, experimental design, and manuscript preparation. ZGG helped with pharmacological assays and experimental design. JL completed the mass spectrometry characterization of dendrimer derivatives. AS helped with the fluorescent microscopy. KAJ assisted with the experimental design and manuscript preparation.

## Supplementary Material

Additional file 1 Supplementary figures 1–4. Figure S1: ESI (+) MS of G3 and G2.5 Dendrimers. Figure S2: ESI (+) MS of Compounds **12 **and **13**. Figure S3: ESI (+) MS of Compounds **16 **and **17**. Figure S4: Light and Fluorescent Microscopy of CHO or CHO A_3 _cells with Compound **15**. The cells were plated 24 h prior to the experiment. The cells were incubated for 1 h with the 10 μM of **15 **and imaged with light and fluorescent microscopy. A. CHO A_3 _cells, fluorescent image B. CHO A_3 _cells, light image C. CHO cells, fluorescent image D. CHO cells, light image. Click here for file
