# Supplementary material for: Enhanced A3 adenosine receptor selectivity of multivalent nucleoside-dendrimer conjugates
Source: J Nanobiotechnology. 2008 Oct 23;6:12. doi: 10.1186/1477-3155-6-12 (PMC2582240; doi:10.1186/1477-3155-6-12)

**Figure S1: ESI (+) MS for G3 and G2.5 PAMAM Dendrimer**

G3


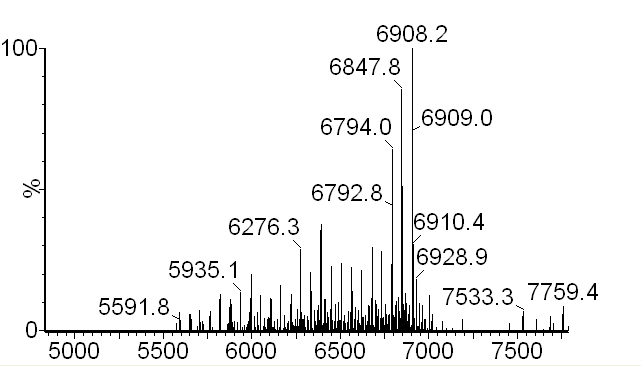


G2.5

**
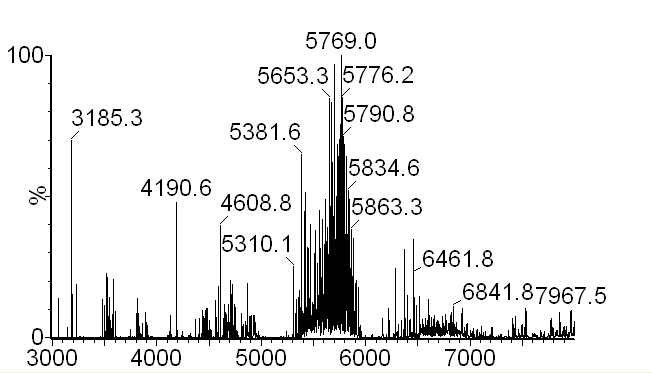
**

**Figure S2: ESI (+) MS of 12 and 13**

**12**

**
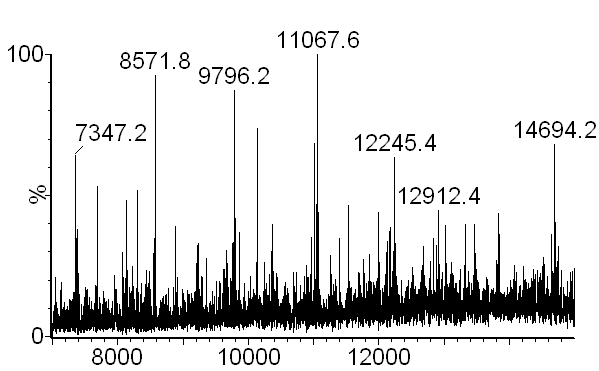
**

**13**

**
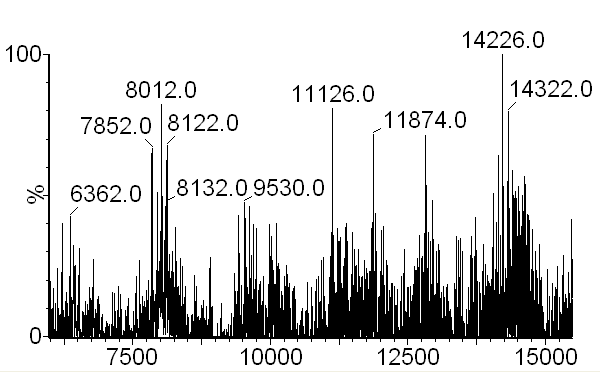
**

**Figure S3: ESI (+) MS of 16 and 17**

**16**

**
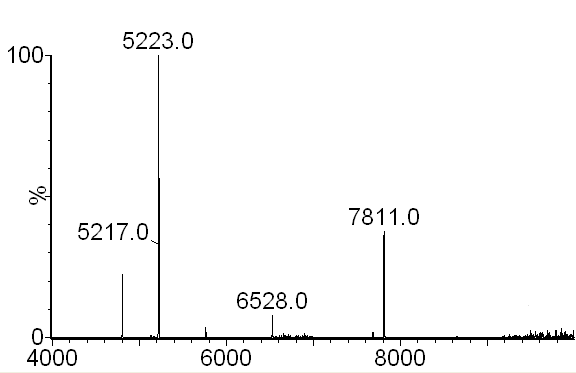
**

**17**

**
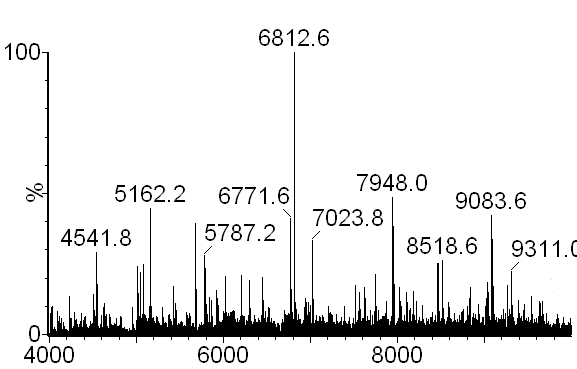
**

Figure S4: Light and Fluorescent Microscopy of CHO or CHO A_3_ cells with Compound **15**.


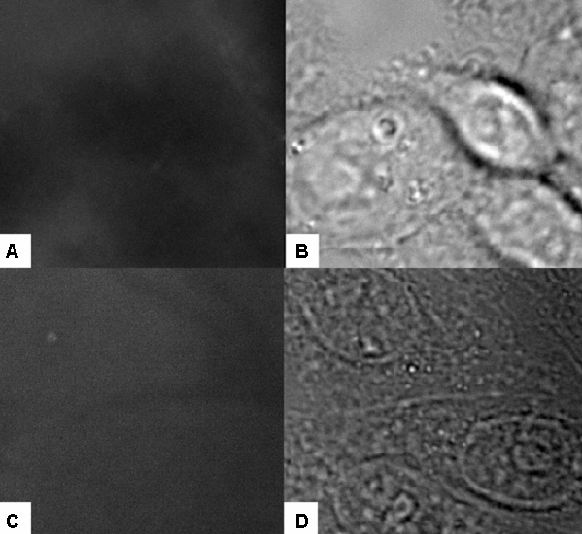

Supplement: Additional file 1 — Supplementary figures 1–4. Figure S1: ESI (+) MS of G3 and G2.5 Dendrimers. Figure S2: ESI (+) MS of Compounds 12 and 13. Figure S3: ESI (+) MS of Compounds 16 and 17. Figure S4: Light and Fluorescent Microscopy of CHO or CHO A3 cells with Compound 15. The cells were plated 24 h prior to the experiment. The cells were incubated for 1 h with the 10 μM of 15 and imaged with light and fluorescent microscopy. A. CHO A3 cells, fluorescent image B. CHO A3 cells, light image C. CHO cells, fluorescent image D. CHO cells, light image. [file 1477-3155-6-12-S1.doc]
